# *NtWRKY-R1*, a Novel Transcription Factor, Integrates IAA and JA Signal Pathway under Topping Damage Stress in *Nicotiana tabacum*

**DOI:** 10.3389/fpls.2017.02263

**Published:** 2018-01-15

**Authors:** Weihuan Jin, Qi Zhou, Yuanfang Wei, Jinmiao Yang, Fengsheng Hao, Zhipeng Cheng, Hongxiang Guo, Weiqun Liu

**Affiliations:** Key Laboratory for Tobacco Cultivation of Tobacco Industry, College of Life Science, Henan Agricultural University, Zhengzhou, China

**Keywords:** *NtWRKY-R1*, topping damage, IAA, JA, signal pathway

## Abstract

Topping damage can induce the nicotine synthesis in tobacco roots, which involves the activation of JA and auxin signal transduction. It remains unclear how these hormone signals are integrated to regulate nicotine synthesis. Here we isolated a transcription factor *NtWRKY-R1* from the group IIe of WRKY family and it had strong negative correlation with the expression of *putrescine N-methyltransferase*, the key enzyme of nicotine synthesis pathway. *NtWRKY-R1* was specifically and highly expressed in tobacco roots, and it contains two transcriptional activity domains in the N- and C-terminal. The promoter region of *NtWRKY-R1* contains two cis-elements which are responding to JA and auxin signals, respectively. Deletion of *NtWRKY-R1* promoter showed that JA and auxin signals were subdued by *NtWRKY-R1*, and the expression of *NtWRKY-R1* was more sensitive to auxin than JA. Furthermore, Yeast two-hybrid experiment demonstrated that NtWRKY-R1 can interact with the actin-binding protein. Our data showed that the intensity of JA and auxin signals can be translated into the expression of *NtWRKY-R1*, which regulates the balance of actin polymerization and depolymerization through binding actin-binding protein, and then regulates the expression of genes related to nicotine synthesis. The results will help us better understand the function of the WRKY-IIe family in the signaling crosstalk of JA and auxin under damage stress.

## Introduction

Nicotine is a secondary metabolite exclusively synthesized in tobacco roots. Topping, removal of the flowering head and adjacent young leaves, is an important cultivating measure for flue-cured tobacco. Many studies have indicated that mechanical damage induces the accumulation of secondary metabolites for enhancing the defense ability including activation of JA and auxin-signaling transduction system (Machado et al., 2013, 2016). Topping damage occurs in over-ground part of plant and changes the hormonal balance including the jasmonate (JA) and auxin (Baldwin et al., 1994), which then leads to the increase of nicotine synthesis in tobacco roots (Hibi et al., 1994; Li et al., 2016). Both JA and auxin are important for plant response to wounding attack (Hibi et al., 1994; Machado et al., 2013; Zhang et al., 2016). The JA signaling pathway is linked to auxin homeostasis through regulating some genes expression (Khan and Stone, 2007; Gutierrez et al., 2012; Hentrich et al., 2013; Loba et al., 2017). It is still unclear the molecular mechanism of JA and auxin signals regulating nicotine synthesis in tobacco.

JA is an important hormone activating plant defense responses to environmental stresses (Liechti and Farmer, 2002). As a component of the long-distance signal-transduction pathway, JA has been implicated to play very important role in systemic signaling between leaves and roots (Han, 2017; Wasternack and Song, 2017), and it is a positive regulator in damage signaling pathway (Baldwin et al., 1994). Herbivore attack can increase JA levels in local and systemic tissues to trigger the biosynthesis of many defensive metabolites including nicotine (Steppuhn et al., 2004; Howe and Jander, 2008). The exogenous JA could induce the expression of key enzyme genes involved in the nicotine biosynthesis (Shoji et al., 2000).

Auxin is a negative regulator in the signaling pathway of nicotine biosynthesis induced by damage (Mason and Mullet, 1990; Mason et al., 1992; DeWald et al., 1994; Shi et al., 2006). It has been reported that high concentration of auxin could inhibit the expression of *putrescine N-methyltransferase* (*NtPMT*) which is the key enzyme of nicotine synthesis pathway (Hibi et al., 1994; Imanishi et al., 1998a,b). The expression level of *NtPMT* was dramatically increased in tobacco Bright Yellow 2 (BY2) cell after addition of MeJA in culture medium, while it was significantly reduced after 2, 4-D treatment (Xu and Timko, 2004). In the presence of auxin, JA did not enhance the accumulation of alkaloids in cell suspension cultures of *Catharanthus roseus* (L.) G. (Vázquez-Flota and De Luca, 1998).

*WRKY* is one of the largest families of transcriptional regulators in plants and the members of this family usually function as repressors or activators involved in different biological processes (Skibbe et al., 2008). The *WRKY* is extremely sensitive to damage stimulation. *WIZZ*, a member of *WRKY*, participated in early stages of the wounding response in tobacco, and it was significantly increased up to the maximum at 5 min after wounding treatment (Seo et al., 1999; Hara et al., 2000). The expression of *AtWRKY6* was increased after wounding and kept in relatively high level in 6 h (Robatzek and Somssich, 2001). *WRKY6* and *WRKY3* were induced by damage of insect bites in the tobacco, and tobacco resistance was improved through increasing JA content (Skibbe et al., 2008). WRKY is also involved in biosynthesis of defensive metabolites. CjWRKY1, a member of group-IIWRKY, acted as a specific and comprehensive regulator in berberine biosynthesis (Kato et al., 2007). PsWRKY increased accumulation of narcotine and papaverine after 5 h of wounding in *Papaver somniferum* seedlings (Mishra et al., 2013).

Although a large number of *WRKY* genes are involved in damage stimulation, the majority of them is still unknown whether playing roles in wounding process. WRKY transcription factors play pivotal roles in regulating many stress responses in plants. However, unraveling their roles in abiotic stress responses has lagged behind that in biotic stresses in tobacco. *WRKY* gene family in tobacco was primarily described as regulator for mechanical damage-induced nicotine synthesis. Research on *NtWRKY* family was mainly concentrated on defense, and it is very limited progress in terms of the *NtWRKYs* involved in the crosstalk of IAA and JA signaling pathway.

Previous study has identified topping responsive proteins in tobacco roots using two-dimensional electrophoresis, and some responsive proteins were reported to involve in the auxin and JA signaling pathways (Fu et al., 2013; Li et al., 2016). The *NtWRKY* transcription factor, *NtWRKY-R1* was identified from tobacco roots before and after topping by suppression subtractive hybridization (SSH) and RNA-Seq analysis (Qi et al., 2012). In this study, the data showed that *NtWRKY-R1* functioned as an integrator of the auxin and JA signaling pathways induced by topping damage, in which the nicotine synthesis was regulated.

## Materials and methods

### Plant material and treatments

Tobacco (*Nicotiana tabacum* K326) plants were grown in greenhouse (28°C/22°C day/night) under a 12-h light/12-h dark cycle, and were randomly divided into four groups. When the first flower of inflorescence came into bloom, the following treatments were performed. (i) The flowering head and adjacent to young leaves of tobacco were removed (topping). (ii) After topping, the lanolin containing 30 μM 1-naphthylacetic acid (NAA) and Tween-20 was immediately applied onto the decapitated stem stumps (topping+NAA). (iii) MeJA (0.8 mM) was sprayed on up-leaves of tobacco without topping; (iv) Tobacco without topping was used as a control. Tobacco roots were harvested at 24 h after treatment. These samples were immediately frozen in liquid nitrogen and then stored at −80°C until further analysis.

### Cloning and sequence analysis of *NtWRKY-R1*

A WRKY-like EST sequence (HO059652) was found by screening the SSH cDNA library of tobacco roots before and after topping. This EST sequence was used as a probe to perform BLAST in Genbank (https://blast.ncbi.nlm.nih.gov/Blast.cgi), and the target sequences with high identity were arrayed and assembled into a full length cDNA (1,379 bp) with the software of CAP3. The Open Reading Frame (ORF) of the cDNA was predicted by ORF Finder (www.ncbi.nlm.nih.gov), and this gene was named as *NtWRKY-R1* and the coding sequence was amplified by primers NtWRKY-R1-F/R (Supplementary Table 1).

The obtained sequences were analyzed using online bioinformatics tools (http://www.ncbi.nlm.nih.gov). The sequence region was analyzed using the plant cis-acting regulatory element (CARE) database (http://www.dna.affrc.go.jp/PLACE and http://bioinformatics.psb.ugent.be/webtools/plantcare/html/).

### Over-expression of *NtWRKY-R1* in tobacco leaf mesophyll-derived protoplasts

The coding sequence of *NtWRKY-R1* was amplified and cloned into the *BamH*I/*Kpn* I site of binary vector *pROK2* to generate the *pROK2-NtWRKY-R1* constructs. The specific primers for *pROK2-NtWRKY-R1* constructs were NtWRKY-R1-F/R (Supplementary Table 1).

*Nicotiana benthamiana* leaves were chopped into approximately 0.5 mm strips and immediately transferred into 0.6 M mannitol for 10 min in the dark, and then incubated in an enzyme solution (1.5% Cellulase R10, 0.4% Macerozyme R-10, 0.6 M mannitol, 10 mM MES at pH 5.7, 10 mM CaCl_2_ and 0.1% BSA) for 4–5 h in the dark with gentle shaking (60–80 rpm). After the enzymatic digestion, an equal volume of W5 solution (154 mM NaCl, 125 mM CaCl_2_, 5 mM KCl, and 2 mM MES at pH 5.7) was added and shook for 10 sec. Protoplasts were released by filtering through 40 μm nylon meshes into round bottom tubes with 3–5 washes of the strips using W5 solution. The pellets were collected by centrifugation at 1,500 rpm for 3 min. After washing once with W5 solution, the protoplasts pellet were then resuspended in MMG solution (0.4 M mannitol, 15 mM MgCl_2_ and 4 mM MES at pH 5.7) at a concentration of 2 × 10^6^ cells mL^−1^, and determined by a hematocytometer.

The constructs *pROK2-NtWRKY-R1* and the control vector *pROK2* were transfected in the protoplasts by PEG methods as described previously (Yoo et al., 2007). In briefly, 15 μg of plasmid DNA was mixed with 200 μL protoplasts, and then added 220 μL freshly prepared PEG solution (40% PEG 4000). The mixture was incubated at room temperature for 10–20 min in the dark. After incubation, 1000 μL W5 solution was added slowly and inverted the tube gently, and the protoplasts were collected by centrifugation at 1,500 rpm for 3 min. The protoplasts were resuspended gently in 1 mL WI solution (0.5 M mannitol, 20 mM KCl, and 4 mM MES at pH 5.7), and then were cultured in illuminating incubator for 6–16 h. After harvested and quick frozen with liquid nitrogen, the transformed protoplasts were used for transient expression analysis.

### Expression analysis

Total RNA was isolated from tobacco tissues and protoplasts using TRIzol reagent (Invitrogen, Carlsbad, CA, USA). The reverse transcription reactions were performed with One Step Prime Script RT-PCR Kit (Perfect Real Time). TaKaRa SYBR Premix Ex TaqTM II (Perfect Real Time) was used for quantitative real time PCR on a Bio-Rad IQ5 Real-Time PCR Detection System. *NtActin* was used as internal control. Relative amounts of mRNA were determined with the Cycle threshold (Ct) method. Three replicates were performed for each sample. Fold change was determined using the 2^−ΔΔ^Ct method and error bars represented the standard deviation (*SD*) of the mean. AVEDEV and Student's *t*-tests were used to determine *SD* of the mean. The gene specific primers were NtIAA13-F/R, NtPMT-F/R, NtActin-F/R and qNtWRKY-R-F/R (Supplementary Table 1).

### Subcellular localization

To determine the subcellular localization of NtWRKY-R1, the coding sequence of *NtWRKY-R1* without stop codon was cloned into *BamH*I and *Kpn*I sites of the *pCAMBIA1300* vector to generate constructs *Pro35S: WRKY-R1-GFP. Pro35S: GFP* was used as negative control. K326 tobacco leaves were transformed by injection of *Agrobacterium tumefaciens* GV3130 containing different combinations of *Pro35S: WRKY-R-GFP* and *Pro35S: GFP* plasmids. The epithelial cells of the mesophyll were observed for transient expression of GFP with FV 1000 confocal microscope (Olympus, Tokyo, Japan) at 24 h after transformation. The gene-specific primers were NtWRKY-R1-GF/GR (Supplementary Table 1).

### Cloning of *NtWRKY-R1* promoter

The 2,052-bp promoter sequence of *WRKY-R* was amplified with the method of chromosome walking described in manufacturer instructions for the Advantage 2 PCR Kit (Clontech). The primers pWRKY-R1-outer/inner and the adapter primers (AP1 and AP2) were designed from the gene sequences that were previously obtained. The promoter of *NtWRKY-R1* was cloned into the *pMD18-T* vector (TaKaRa, Dalian, China). The promoter sequence of *NtWRKY-R1* was cloned into *BamH*I and *EcoR*I sites of the *pB1121* vector to generate *pB1121-1803UTR, pB1121-1387UTR, pB1121-793UTR*, and *pB1121-386UTR* constructs, respectively. These constructs were transformed into BY2 cell through *A. tumefaciens* GV3130 as described previously (Navarre et al., 2006). Transgenic calli were selected on MS medium containing 100 mg/L of kanamycin and 25 μg/mL hygromycin. The four independent transgenic BY2 cell lines were treated with 2,4-Dichlorophenoxyacetic acid (2,4-D; 0.5 μM) and MeJA (25 μM), and then incubated at 28°C for 12 h to determine GUS activity. The specific primers for *pB1121-1803UTR, pB1121-1387UTR, pB1121-793UTR*, and *pB1121-386UTR* constructs were 1803UTR-F/Pro-R, 1387UTR-F/Pro-R, 793UTR-F/Pro-R, and 386UTR-F/Pro-R, respectively (Supplementary Table 1).

### Yeast two-hybrid

Yeast two-hybrid assays were performed according to the protocol (Matchmaker Gold Yeast Two-Hybrid manual, clontech). For testing autoactivation, the *NtWRKY-R1* was cloned into *Nde*I and *BamH*I sites of the *pGBKT7* vector to generate *pGBKT7-W1, pGBKT7-W2, pGBKT7-W3, pGBKT7-W4, pGBKT7-W5, pGBKT7-W6*, and *pGBKT7-W7*. The constructs were transformed into the yeast strain AH109. The specific primers for *pGBKT7-W1, pGBKT7-W2, pGBKT7-W3, pGBKT7-W4, pGBKT7-W5, pGBKT7-W6*, and *pGBKT7-W7* were W1-F/R, W2-F/R, W3-F/R, W4-F/R, W2-F/W3-R, W3-F/W4-R, and W2-F/W4-R, respectively (Supplementary Table 1). The *N. benthamiana* normalized library was constructed into *pGADT7* plasmid and transformed into yeast strain Y187. The fragment of *NtWRKY-R1* coding sequence (226–675 bp) was cloned into the *pGBKT7* vector and transformed into the yeast strain AH109 harboring *pGBKT7-W5* used as the prey to screen the *N. benthamiana* normalized library. The mating and screening were performed as described in the protocol. The coding sequence of *ABP* was cloned into *Nde*I and *BamH*I sites of *pGADT7* plasmids to generate the *pGADT7-ABP* constructs. The plasmid *pGBKT7-W5* and *pGADT7-ABP* were co-transformed into yeast strain AH109 to confirm the interaction. The specific primers for *pGADT7-ABP* were NtABP-F/R (Supplementary Table 1).

## Results

### Expression of *NtWRKY-R1* is regulated by JA and auxin signals

**Figure 1:**
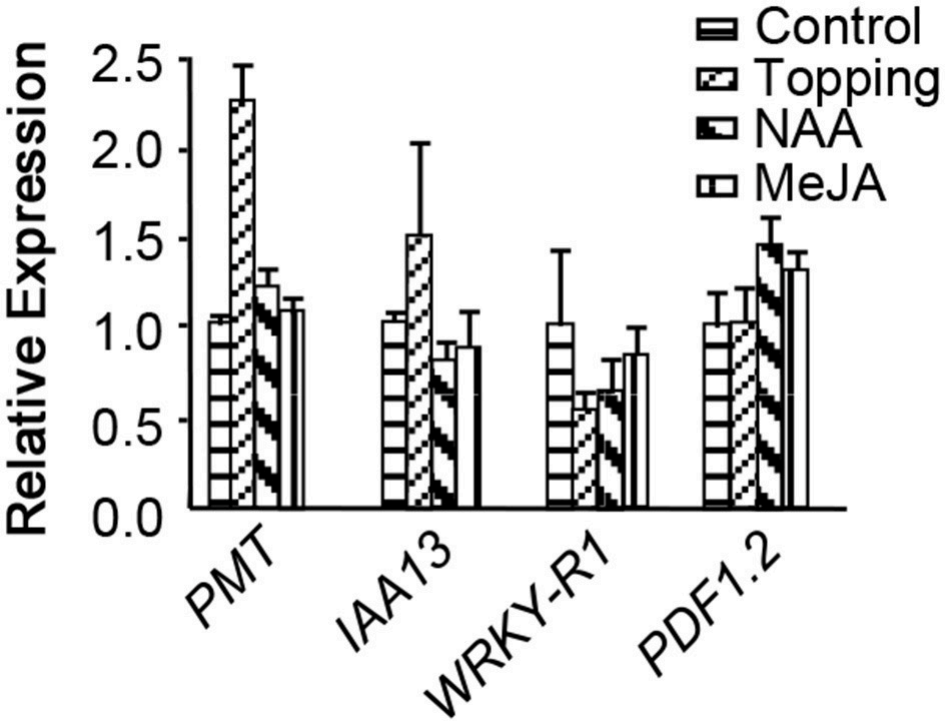
The expression of *PMT, IAA13, NtWRKY-R1*, and *PDF1.2* in response to MeJA, NAA, and topping. Values are given as means ± *SD* relative to the values. The respective control expression levels set at 1.0.

To understand whether *NtWRKY-R1* participates in JA- and auxin-mediated signal transduction and involves in topping damage-induced nicotine biosynthesis, the expression of *NtWRKY-R1, PMT, IAA13*, and *PDF1.2* were determined in tobacco roots at 24 h after topping by qRT-PCR (Figure 1). *PMT* is the enzyme catalyzing the first committed step in nicotine biosynthesis (Steppuhn et al., 2004). *PDF1.2* is commonly used as a marker for characterization of the JA-dependent defense responses (Manners et al., 1998; Penninckx et al., 1998). *IAA13* is an early auxin-responsive gene (Falkenberg et al., 2008). Compared to no-topping treatment, *PMT* was significantly up-regulated, but *NtWRKY-R1* was significantly down-regulated after topping (Figure 1), indicating that NtWRKY-R1 is negative regulator in nicotine biosynthesis. Topping damage can trigger the JA signaling (Singh et al., 2015) and improve the auxin synthesis in root tip tissues due to removal of the auxin source in over-ground part (Fu et al., 2013), therefore, it can explain why the expression level of *PDF1.2* and *IAA13* was up-regulated after topping.

To further investigate the relationship between *NtWRKY-R1* and the signaling pathway of JA and auxin after topping damage, the response of *NtWRKY-R1* to NAA and JA was analyzed (Figure 1). When NAA was applied on decapitated stem stumps, the expression of *NtWRKY-R1* was strongly suppressed. The same effect was observed after spraying with MeJA on the young leaves of tobacco without topping, but the sensitivity of *NtWRKY-R1* to NAA was much greater than JA (Figure 1). It is interesting that the expression level of *NtWRKY-R1* in the two treatments was much higher than topping treatment, and that the *PMT* expression has no obviously difference between the two treatments and no-topping treatment. These results suggested that JA- and auxin-signals were trigged by topping and then mediated the regulation of nicotine biosynthesis.

### Cloning and characterization of *NtWRKY-R1*

**Figure 2:**
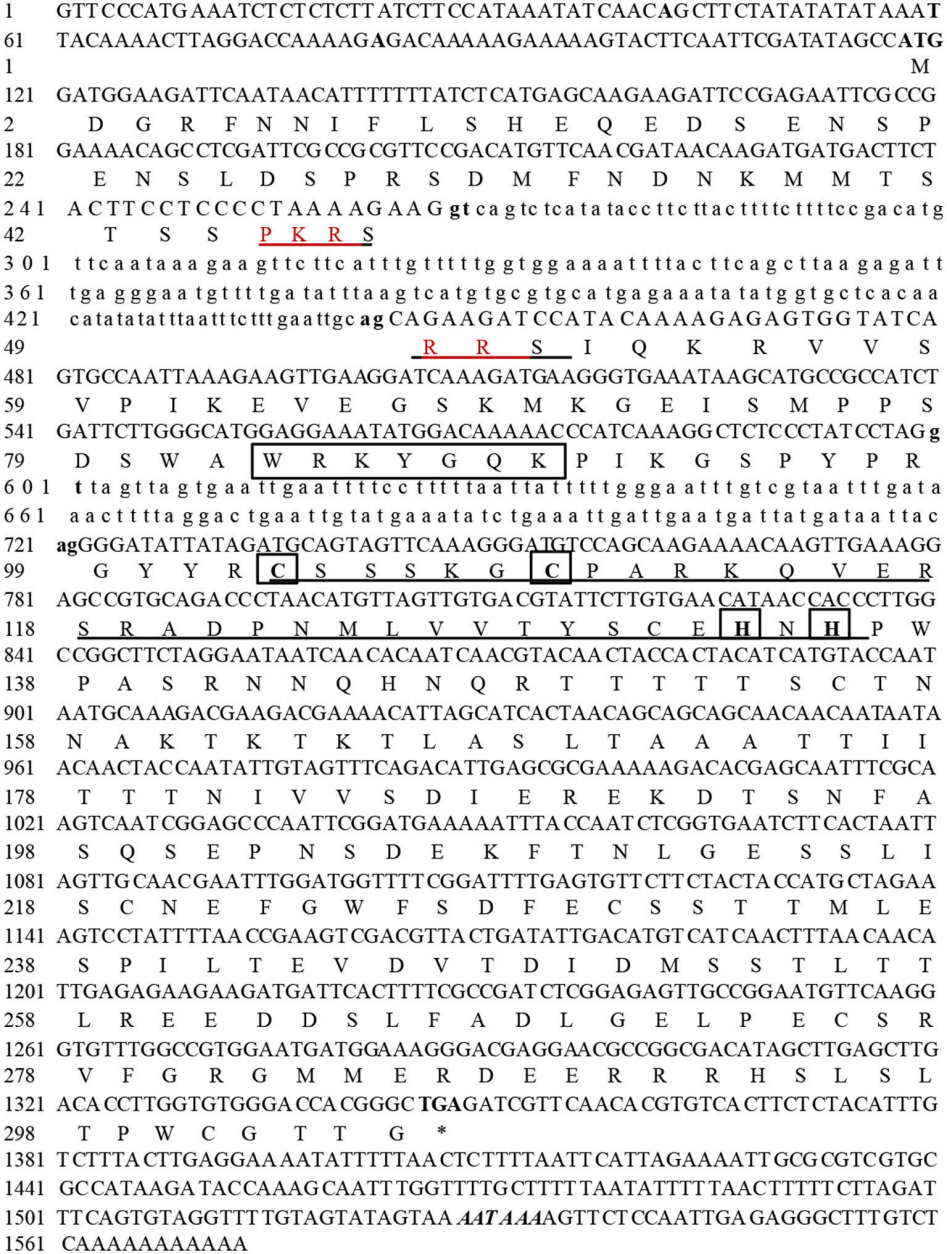
The full-length cDNA, intron, and deduced amino acid sequences of *NtWRKY-R1* gene. The exons sequence is indicated in capital letter and the intron is indicated in lowercase. The start codon (ATG), the stop codon (TAG), and a putative exon-intron splicing sites (gt/ag) are shown by bold letters. The putative nuclear localization signal is underlined. The WRKY domain and zinc finger motif (CX5CX23HX1H) are marked by square brackets.

**Figure 3:**
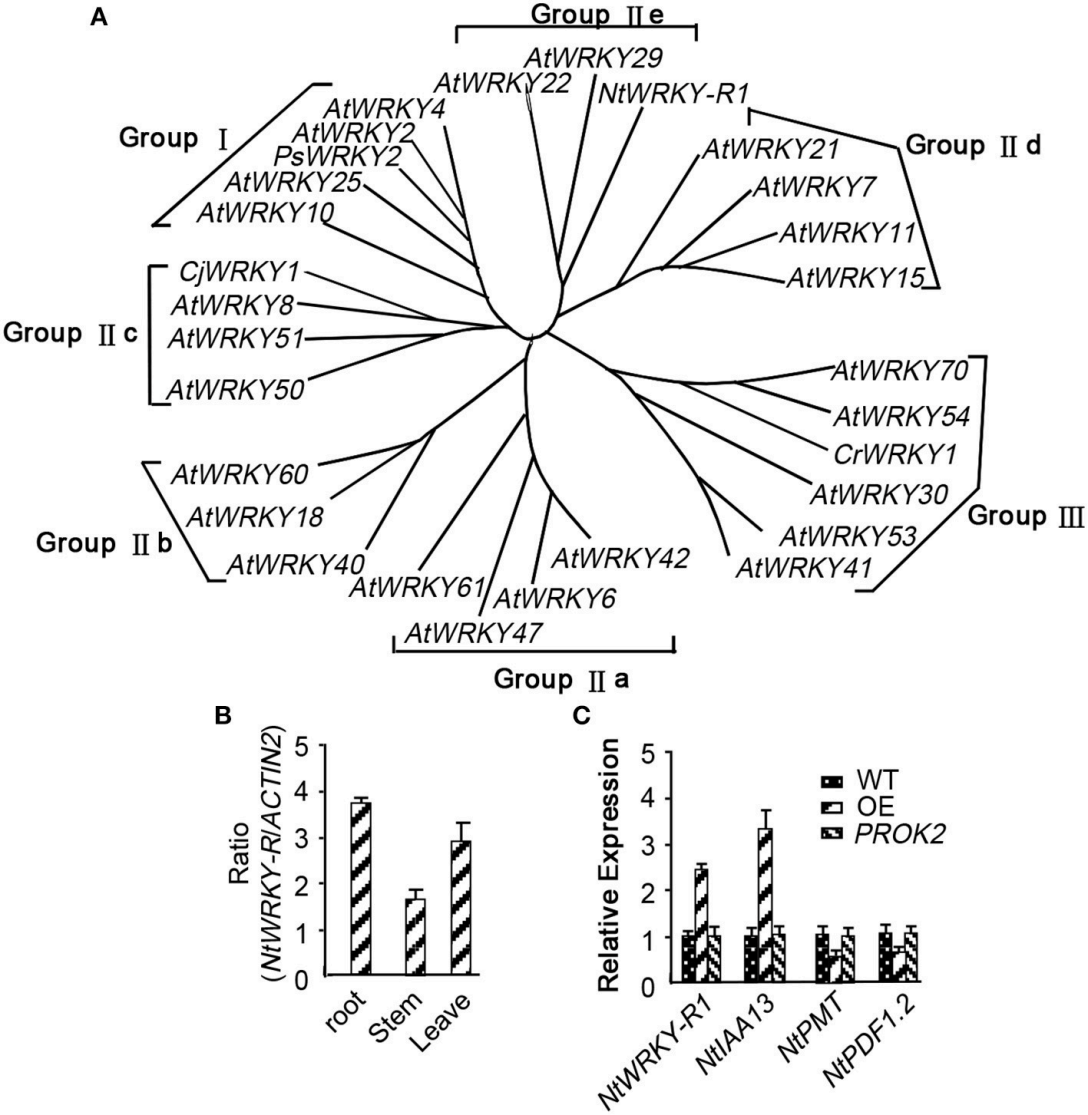
The phylogenetic analysis and expression pattern identification of *NtWRKY-R1*. **(A)** The phylogenetic analysis of *NtWRKY-R1*. **(B)** RT-PCR analysis of *NtWRKY-R1* gene expression in the indicated *Nicotiana tabacum* tissues. Total RNA was isolated from roots, stems and leaves. Data were means ± *SD* of three biological replicates. **(C)** The effect of over-expressed *NtWRKY-R1* on the expression of *PMT, IAA13*, and *PDF1.2*. WT is tobacco leaf mesophyll-derived protoplasts. OE is tobacco leaf mesophyll-derived protoplasts transformed by *NtWRKY-R1* with the 35S promoter. *PROK2* is tobacco leaf mesophyll-derived protoplasts transformed by empty vector.

Although the response of *NtWRKY-R1* to auxin and JA has been showed in tobacco topping, it is unclear that the function of *NtWRKY-R1* as a node of convergence for JA- and auxin-mediated signaling. To further verify the relationship between the expression of *NtWRKY-R1* and the signal transduction pathway of auxin and JA, the full-length cDNA of *NtWRKY-R1* was amplified by RT-PCR, and the sequence was analyzed with bioinformatics. The EST sequence of *NtWRKY-R1* from SSH library was used as inquiring probe to blast the tobacco EST database. After spliced through overlap extension, a cDNA of 1,379 bp was obtained, containing a 915 bp ORF which encodes a putative protein of 305 amino acids. The *NtWRKY-R1* gene contained two introns and three exons, and there were 153 bp 5′-end and 311 bp 3′-end UTR (Figure 2). In the end, the *NtWRKY-R1* gene was amplified from tobacco roots. Multiple sequence alignment showed that NtWRKY-R1 was highly conserved and contained two conservative motifs of WRKY family, WRKYGQK and C_2_H_2_ (Figure 2). The phylogenetic analysis showed that it had been assigned to group IIe of the WRKY transcription factor family (Figure 3A). The tissue specific expression assay showed that *NtWRKY-R1* had the highest expression in roots (Figure 3B).

### *NtWRKY-R1* mediates JA and auxin signaling transduction

Both JA and auxin signaling pathways were induced by topping damage. To understand whether *NtWRKY-R1* involved in the crosstalk of JA and auxin signaling, the transient gene expression system using mesophyll protoplasts was performed. It had been reported that *PMT, IAA13*, and *PDF1.2* can be expressed in leaves. Therefore, mesophyll cell from young leaves, subjected to nutrients and energy starvation for 24 h, was used. At 6–8 h of incubation after the *NtWRKY-R1* was transformed to the tobacco leaf mesophyll-derived protoplasts, the expression level of *PDF1.2, IAA13*, and *PMT* were determined by qPCR (Figure 3C). The expression of *IAA13* was increased and the expression of *PDF1.2* was decreased, while the decrease in *PMT* expression can be evidently observed, indicating that the *NtWRKY-R1* is involved in the homeostasis between JA and auxin signaling conduction.

### *NtWRKY-R1* is localized in nuclear bodies

**Figure 4:**
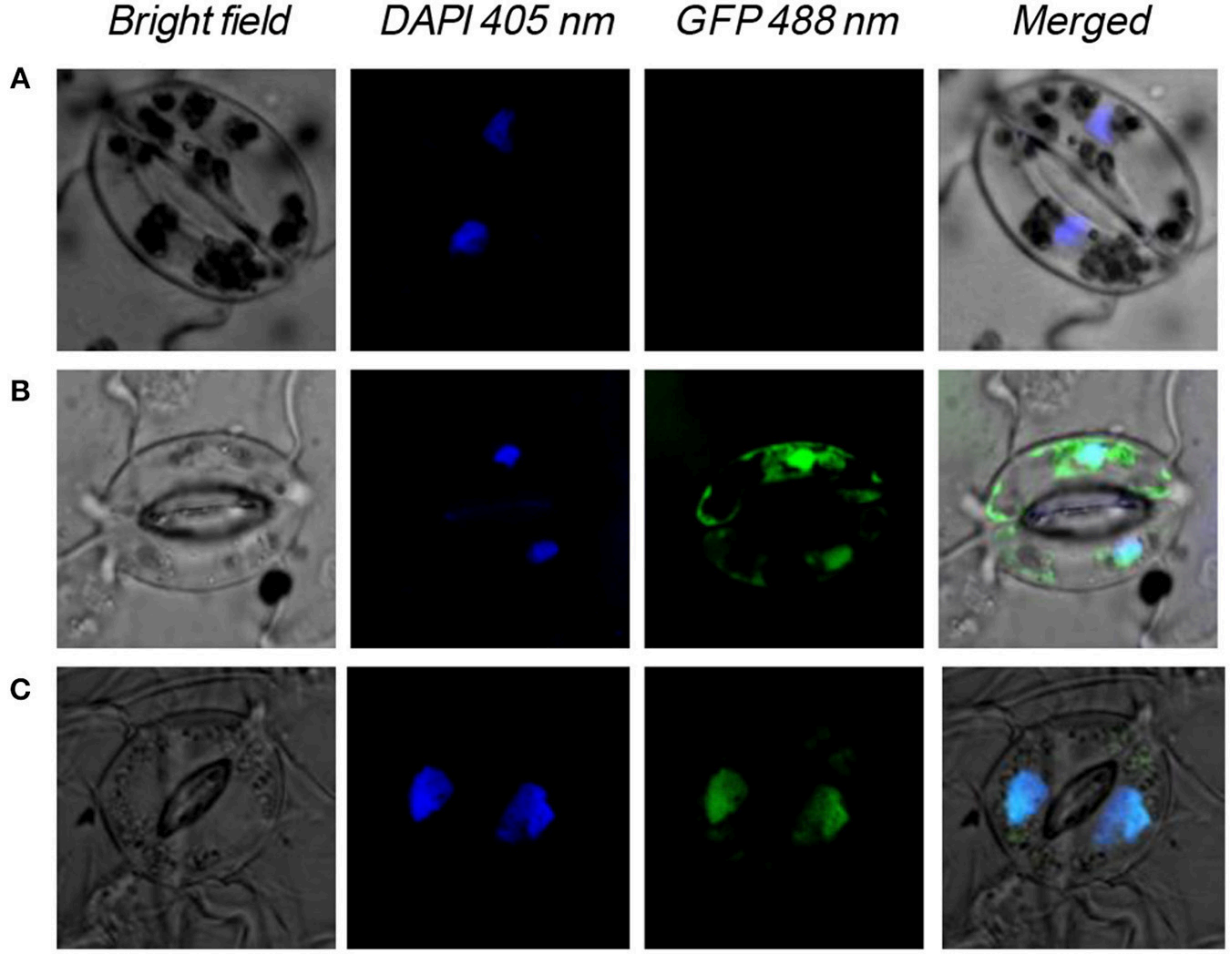
Nuclear localization of *NtWRKY-R1* in tobacco cells. **(A)** The control plant, **(B)** The transformed plant with p*S1300:GFP* vector, **(C)** the transformed plant with p*S1300:NtWRKY-R1:GFP*. The emission wave lengths of bright field, DAPI and GFP are 510, 405, and 488 nm, respectively.

To determine whether NtWRKY-R1 functions as a transcription factor in cell nucleus, its subcellular localization was performed. *NtWRKY-R1* was linked to *GFP* reporter gene to generate fusion construct, and then it was transformed into *A. tumefaciens* GV3130. Bacterial suspension was directly infiltrated into intercellular spaces of tobacco leaves using a syringe without needle. At 24 h after transformation, the desired infiltrated leaves were observed for transient expression analysis with confocal laser-scanning microscope. In the cells which were transformed with constructs *Pro35S:WRKY-R1-GFP*, NtWRKY-R1-GFP fusion protein was exclusively localized in the nucleus (Figure 4), whereas GFP protein was observed throughout the entire cells which were transformed with constructs *Pro35S:GFP*. These results demonstrated that NtWRKY-R1 was localized in the nucleus.

### NtWRKY-R1 functions as the integrator of JA and auxin signaling

**Figure 5:**
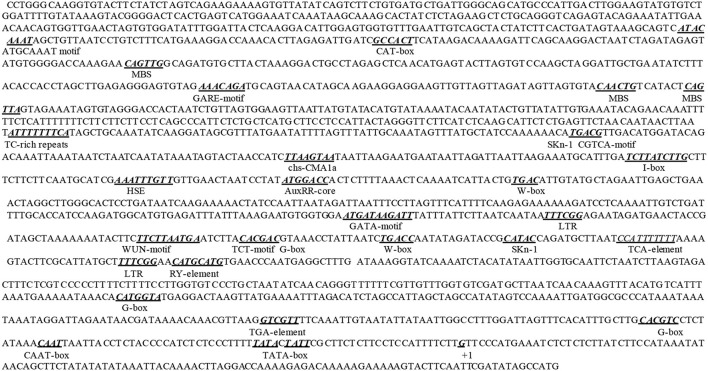
The bioinformatics analysis of *NtWRKY-R1* promoter. TC-rich repeats: *Nicotiana tabacum* cis-acting element involved in defense and stress response; TGA-element: *Brassica oleracea* auxin-responsive element (−132 ~ −137); TCA-element: *Nicotiana tabacum* cis-acting element involved in salicylic acid response; LTR: *Hordeum vulgare* cis-acting element involved in low-temperature response; AuxRR-core: *Nicotiana tabacum* cis-acting regulatory element involved in auxin response (−855 ~ −862); CGTCA-motif: *Hordeum vulgare* cis-acting regulatory element involved in the MeJA-response (−1,025 ~ −1,030); GARE-motif, *Brassica oleracea* gibberellin-responsive element; ATGCAAAT motif, cis-acting regulatory element associated to the TGAGTCA motif; HSE, cis-acting element involved in heat stress response; G-box, I-box, TCT-motif, chs-CMA part of a light responsive element; MBS, MYB binding site involved in drought-inducibility; SKn-1, cis-acting regulatory element required for endosperm expression; CAT-box, cis-acting regulatory element related to meristem expression; RY-element, cis-acting regulatory element involved in seed-specific regulation; WUN-motif, wound-responsive element.

**Figure 6:**
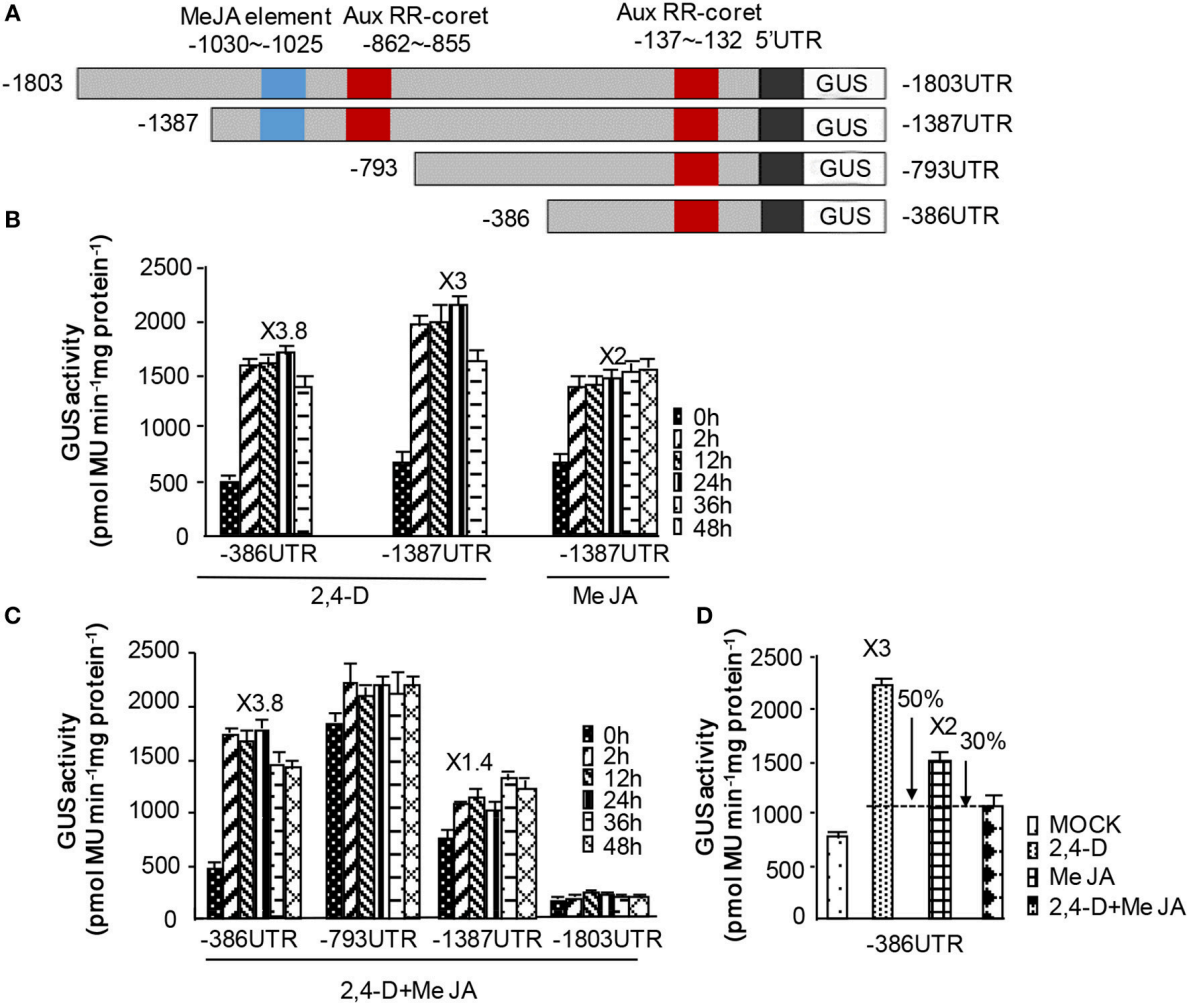
The NtWRKY-R1 integrated the auxin and JA signaling. **(A)** The promoter fragments of *NtWRKY-R1*. This promoter contains the MeJA and AuxRR elements. Schematic diagrams of *NtWRKY-R1* promoter and its deletions were shown. **(B–D)** The GUS activities of the deleted and complete promoters were monitored. The deleted *NtWRKY-R1* promoters were fused to *GUS* reporter gene, and then transformed into BY2 cell. We measured GUS activities at 0, 2, 12, 24, 36, and 48 h after treatment with JA and/or auxin.

To investigate the integrating function of NtWRKY-R1 in the JA and auxin signaling pathway, its promoter with −1,803 bp in length was obtained from *N. tabacum* roots. One JA and two auxin cis-elements in the promoter of *NtWRKY-R1* were analyzed by PlantCARE databases (Figure 5). The three of promoter derivatives (5′ deletions −1,387, −793, −386 bp) and full length promoter −1,803 bp of *NtWRKY-R1* were fused to the *GUS* reporter gene, and then transformed to BY2 cell, respectively. After incubation with different concentrations of 2,4-D and MeJA, we observed the strongest GUS activity in the treatment of 0.5 μM 2,4-D or 25 μM MeJA, so this concentration of 2,4-D and MeJA was used for the following experimental analysis (Supplementary Figure 1). After addition of MeJA, GUS activity in BY2 cell expressing 1,387 bp *NtWRKY-R1* promoter was increased 2-fold compared to control. When BY2 cells harboring −1,387 and −386 bp *NtWRKY-R1*-promoter were respectively incubated with 2,4-D, GUS activities significantly increased, but BY2 cell with −386 bp *NtWRKY-R1* promoter was more sensitive to 2,4-D than −1,387 bp (Figure 6). The reason may be that the JA cis-element in the 5′-proximal region of auxin cis-element was deleted, which eliminated the influence of JA cis-element adjacent to auxin cis-element. When incubation with 2,4-D and MeJA, the GUS activity in BY2 cell harboring −1,387 bp promoter decreased by 1.6-fold compared with the only 2,4-D treatment, and decreased by 0.4-fold compared with the only MeJA treatment. But there was no significantly change in BY2 cell harboring −386 bp (Figure 6). These results indicated that the NtWRKY-R1 is responsible for eliminating the signal intensity in crosstalk of auxin and JA signaling pathway induced by wounding damage, in which the expression level of *NtWRKY-R1* was regulated.

### Identification of the interactors of *NtWRKY-R1* by yeast two-hybrid

**Figure 7:**
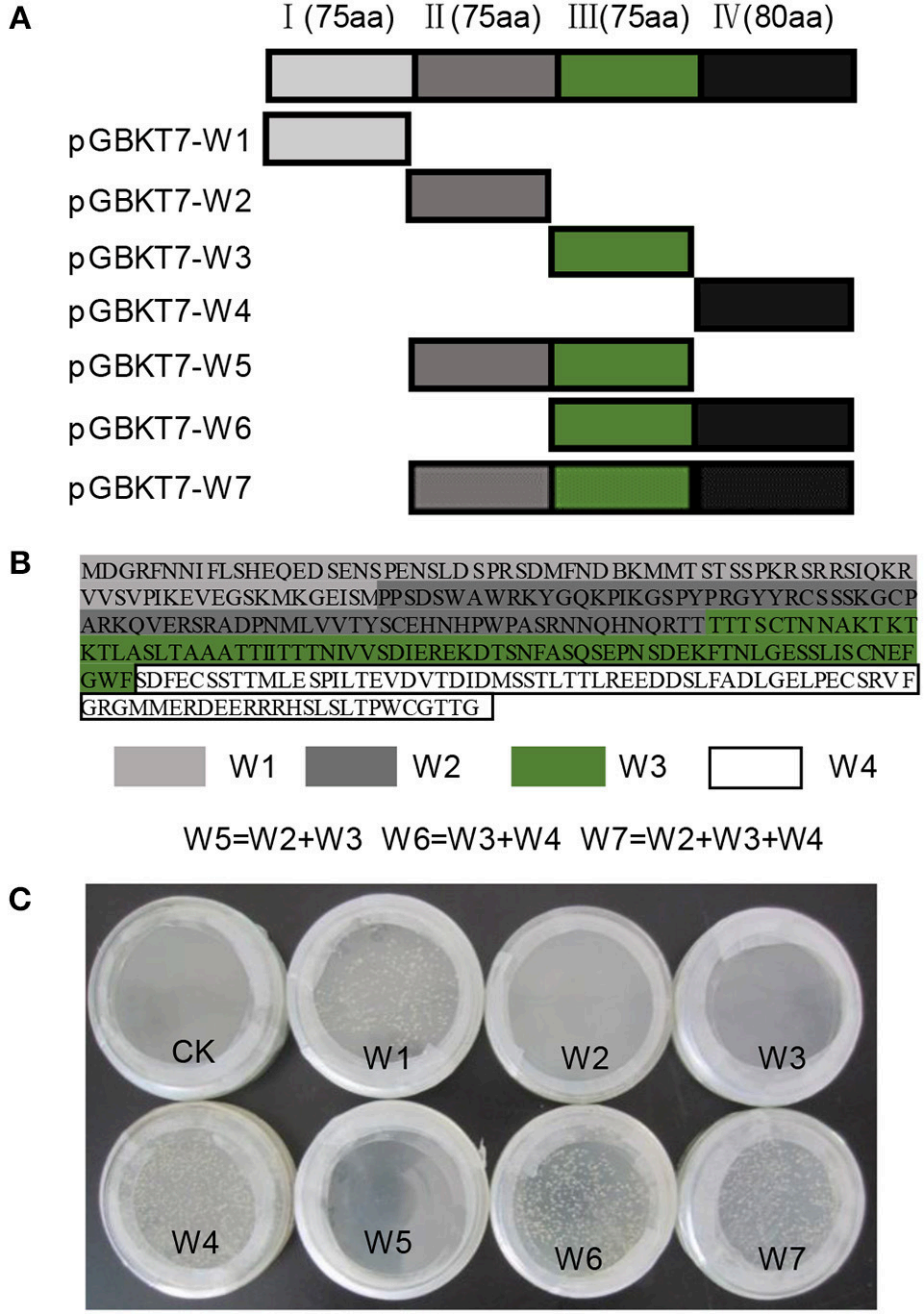
The transcriptional activation analysis of NtWRKY-R1. **(A,B)** the protein fragment of NtWRKY-R1. This protein contains the W1, W2, W3, and W4 elements. Schematic diagrams of NtWRKY-R1 protein and its deletions were shown. **(C)** Testing autoactivation of NtWRKY-R1 by yeast two hybrid systems. The bait NtWRKY-R1 and its different elements were transformed into YH109 yeast stain to confirm autoactivation in the absence of a prey protein.

**Figure 8:**
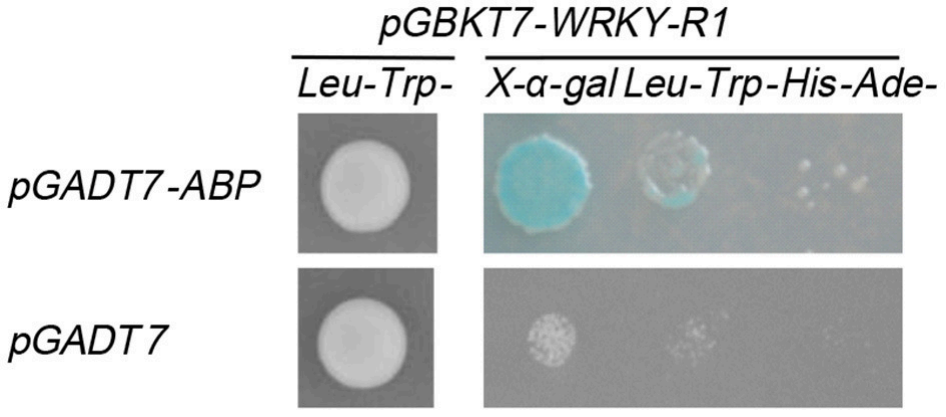
NtWRKY-R1 strongly interacted with ABP.

Some investigations suggested that WRKY proteins functioned through forming protein complexes with other interactors (Xu et al., 2004; Miao and Zentgraf, 2007). To find out the interactors of NtWRKY-R1, we performed a yeast two-hybrid experiment, and the results showed that there was apparent autoactivation of the full-length NtWRKY-R1 (Figure 7). To identify the function region of the transcriptional activation, the assay of several mutants with deletion was performed. The 305 amino acid residues of NtWRKY-R1 were divided into four subsections (Figures 7A,B), then each was fused to the bait vector and transformed to the AH109 yeast strain, respectively. The results showed that the activity region of transcriptional activation of NtWRKY-R1 located in the 80 amino acids of N terminus and the 75 amino acids of C terminus (Figure 7C). The residual 150 amino acids (II and III region) was fused to *pGBKT7* vector and then transformed to the *AH109* yeast strain. After screening a *N. benthamiana* cDNA expression library, 15 positive colonies were identified as potential interactors. Sequencing analysis showed that 10 colonies can match the proteins sequence. Of these colonies, about forty percent are actin-binding protein (ABP)/actin depolymerizing factor (ADF). To confirm the interaction between NtWRKY-R1 and ABP, we constructed the *pGADT7-ABP* and *pGBKT7-WRKY* truncated (amino acids 76–225). The interaction was detected by yeast growth on quadruple dropout plates. As shown in Figure 8, the truncation *WRKY* was able to interact with ABP.

## Discussion

Extensive studies demonstrated that nicotine synthesis can be induced by topping damage, in which JA functions as an important positive regulator (Baldwin et al., 1994), while auxin acts as the negative regulator (Solt, 1957; Shi et al., 2006; Li et al., 2007; Wang et al., 2008). Application of auxin on the damaged leaves could inhibit the induced-nicotine synthesis and reduce the JA content in leaves. If smearing the auxin on non-damaged leaves, the JA content did not change in damage leaves. Therefore, it is considered that the auxin inhibits nicotine syntheses induced by damage through the JA signaling pathway (Baldwin et al., 1994). In recent years, it was found that JA and auxin had very similar signal transmission mechanism, and there were the shared components in their signal transmission pathway (Tiryaki and Staswick, 2002). Our experimental data showed that NtWRKY-R1 played an important role in the crosstalk between JA and auxin signaling pathway induced by topping damage (Figure 1). The indirect and direct responses to JA and auxin signals are compromised in plant, in which the intensity of JA and auxin signaling can be subdued through the NtWRKY-R1. When the exogenous NAA was applied on the damage site, the down-regulation of *NtWRKY-R1* after topping can be counteracted. Meanwhile, the expression level of *PDF1.2* was also suppressed (Figure 1), indicating that the auxin interfered JA biosynthesis. However, how JA and IAA collaboratively respond to topping stimulation and regulate nicotine biosynthesis is still unclear.

WRKY transcription factors can form convergent part of signaling network that modulates many biological processes (Rushton et al., 2010). Although WRKY transcription factors are structurally conversed, the cis-element and other assistant factors could be entitled to WRKY members with diverse biological activities (Skibbe et al., 2008). For example, *CrWRKY1* is preferentially expressed in roots and induced by several phytohormones, such as jasmonate, gibberellic acid, and ethylene (Suttipanta et al., 2011). Apart from the potential involvement in regulating plant defense responses, nearly nothing is known about the role of WRKY transcription factors in other plant signaling processes. In this study, we isolated *NtWRKY-R1*, which has high tissue-specific expression in tobacco roots (Figure 3B). The NtWRKY-R1 was located in the nucleus and functioned as a transcriptional repressor (Figures 4, 6). A phylogenetic analysis revealed that *NtWRKY-R1* had a close relationship with *AtWRKY22* and *AtWRKY27*, belonging to group IIe of *WRKY* family (Figure 3A). *In vivo* assay showed that there were two transcriptional activity domains in the N- and C-terminal of the NtWRKY-R1 (Figure 7). Although group IIe of WRKY family still needs to be studied in detail, a common feature of many domains affecting transcription is the predominance of certain amino acids, including rich region of S-Island-T-Island, E in N-terminal and acidic amino acids in C-terminal. These domains play a pivotal role for regulation of activation (Triezenberg, 1995; Hanna-Rose and Hansen, 1996), which further supports the activation domains of NtWRKY-R1 in N- and C-terminus (Figure 2).

There is diversity in the response of WRKYs to the hormone (Ramamoorthy et al., 2008). Recently, it was reported that WRKY57 in *Arabidopsis* was responsible for crosstalk of JA- and IAA-mediated leave senescence, and that JA24/8 and IAA290 protein competitively interacted with WRKY57 protein (Jiang et al., 2014). In our study, the experiment of deleting *NtWRKY-R1* promoter directly demonstrated that JA and auxin signal was subdued by NtWRKY-R1, and the expression of NtWRKY-R was more sensitive to auxin than JA. When treated with NAA and MeJA at the same time, the subduction effect of NAA was more obvious than JA (Figure 6). These data indicated that NtWRKY-R1 can mediate the crosstalk of JA and IAA signaling pathway.

Yeast two-hybrid experiment further showed that NtWRKY-R1 could interact with the ABP/ADF (Figure 8). The ABP/ADF is considered as primary regulatory factor for the reorganization of the actin cytoskeleton (Maciver and Hussey, 2002; Zhao et al., 2015), which is involved in various cellular and developmental activities (Ketelaar et al., 2004; Kandasamy et al., 2007), as well as cell signaling in response to biotic and abiotic stresses (Hussey et al., 2002). The actin in the nucleus, as a structural protein, participates in the formation of the nuclear skeleton and chromatin skeleton, binds ADF to regulate chromatin structure and gene transcription, mRNA processing and transportation (Burgos-Rivera et al., 2008; Zheng et al., 2013; Fu et al., 2014; Henty-Ridilla et al., 2014). *HbADF* was suggested to be possibly involved in the latex regulation and wound plugging in *Hevea brasiliensis* (Long et al., 2015; Khatun et al., 2016). It had been proved that ADF genes can be induced by low temperature, damage, auxin, and JA in *Arabidopsis*, tobacco and wheat (Vidali et al., 2009; Durst et al., 2013; Fu et al., 2014; Khatun et al., 2016).

In this paper, we isolated *NtWRKY-R1* from tobacco roots, and it was found to medicate the crosstalk of JA and auxin signaling pathway. The intensity of JA and auxin signals can be translated into the expression of *NtWRKY-R1*, which probably regulates the balance of actin polymerization and depolymerization through binding with ABP/ADF, and then regulate the expression of genes related to nicotine synthesis. These results will give us a new clue to understand the function of the WRKY-IIe family in the signaling crosstalk of JA and IAA under damage stress.

## Author contributions

This research was designed by HG and WL. The experiments were performed by WJ, QZ, YW, JY, FH, and ZC, and the data were analyzed by WJ, HG, and WL. The manuscript was written by WJ, HG, and WL.

### Conflict of interest statement

The authors declare that the research was conducted in the absence of any commercial or financial relationships that could be construed as a potential conflict of interest.

## Abbreviations

PMT: putrescine N-methyltransferase
JA: jasmonate
ABP: actin-binding protein
ADF: actin depolymerizing factor
PDF: plant defensin
IAA13: Auxin-induced protein 13
2,4-D: 2,4-Dichlorophenoxyacetic acid
PEG: polyethylene glycol.
