# Supplementary material for: NtWRKY-R1, a Novel Transcription Factor, Integrates IAA and JA Signal Pathway under Topping Damage Stress in Nicotiana tabacum
Source: Front Plant Sci. 2018 Jan 15;8:2263. doi: 10.3389/fpls.2017.02263 (PMC5775218; doi:10.3389/fpls.2017.02263)
Supplement: Supplementary file 1 [file Table1.DOCX]

**NtWRKY-R1, a novel transcription factor, integrates IAA and JA signal pathway by topping damage in *Nicotiana tabacum***

Weihuan Jin^a^, Qi Zhou^a^, Yuanfang Wei, Jinmiao Yang, Fengsheng Hao, Zhipeng Cheng，Hongxiang Guo^*^,Weiqun Liu^*^

**Key Laboratory of national tobacco cultivating, college of life science, henan agricultural university, Zhengzhou china**

^a^ These authors contributed equally to this work.

* Address correspondence to guohongxiang06@126.com or liuweiqun2004@126.com

Correspondence and requests for materials should be addressed to guohongxiang06@126.com or liuweiqun2004@126.com

**Supplemental Table 1 the primers in the paper**

| **Primer name** | **Primer sequence** |
| --- | --- |
| **Primers for RT-PCR** | |
| qNtWRKY-R1-F | ATGGATGGAAGATTCAAT |
| qNtWRKY-R1-R | CAGCCCGTGGTCCCACAC |
| NtPDF 1.2-F | GCCCTTCAATCTCTTCCAAT |
| NtPDF 1.2-R | TATCAGGAAG ACTTGCAGCG |
| NtIAA13-F | GATTACAATATGGACATGTG |
| NtIAA13-R | TAGCTTTGGC ACCTGTGATC |
| NtPMT-F | GCACTTCTGAACACCTCAAC |
| NtPMT-R | CTGTTGTTCGGATGTCCCAT |
| NtActin-F | ATGGCGGATGGGGAGGACAT |
| NtActin-R | TTAGAAGCATTTGCGGTG |
| **Primers for construction** | |
| NtGRAS-R1-F | GCTAGGATCCCATGGATGGAAGATTCAAT |
| NtGRAS-R1-R | TAGGTACCTCAGCCCGTGGTCCCACAC |
| NtWRKY-R1-GF -GF | GCTAGGATCCCATGGATGGAAGATTCAAT |
| NtWRKY-R1-GF -GR | TAGGTACCGCCCGTGGTCCCACACCA |
| pWRKY-R1-outer | TAGTGTAAAAAGGGAGAGATGGGGTAGAG |
| pWRKY-R1-inner: | GCAAGCAAATGTGAAACTAATCCAAAGGC |
| 386UTR-F | CGGGATCCTAGACTTTCTCGTCCCCCT |
| 793UTR-F | CGGGATCCTTGGGCACTCCTGATAAT |
| 1387UTR-F | CGGGATCCGTAACATAGCAAGAAGGAGG |
| 1803UTR-F | CGGGATCCAGTCATGGAAATCAAATAAGC |
| pro-R | GGAATTCCGGCTATATCGAATTGAAGTAC |
| W1-F | GCAT ATG GATGGAAGATTCAATAAC |
| W1-R | AGGATCCCATGCTTATT TCACCCTTCAT |
| W2-F | GCAT ATG CCGCCATCTGATTCTTGGGCAT |
| W2-R | AGGATCCAGTTGTACGT TGATTGTGTTG |
| W3-F | GCAT ATG ACCACTACATCATGTACCAAT |
| W3-R | AGGATCCAAACCATCCA AATTCGTTGCA |
| W4-F | GCAT ATG TCGGATTTTGAGTGTTCTTCT |
| W4-R | AGGATCCTCAGCCCGTG GTCCCACACC A |
| NtABP-F | GCAT ATGGCTA ATGCAGCGTC TGGGA |
| NtABP-R | AGGATCCCTAGTTGGCG CGGCTCTTAA T |
