# Supplementary material for: NtWRKY-R1, a Novel Transcription Factor, Integrates IAA and JA Signal Pathway under Topping Damage Stress in Nicotiana tabacum
Source: Front Plant Sci. 2018 Jan 15;8:2263. doi: 10.3389/fpls.2017.02263 (PMC5775218; doi:10.3389/fpls.2017.02263)
Supplement: Supplementary file 2 [file Presentation1.PPTX]

## Slide 1
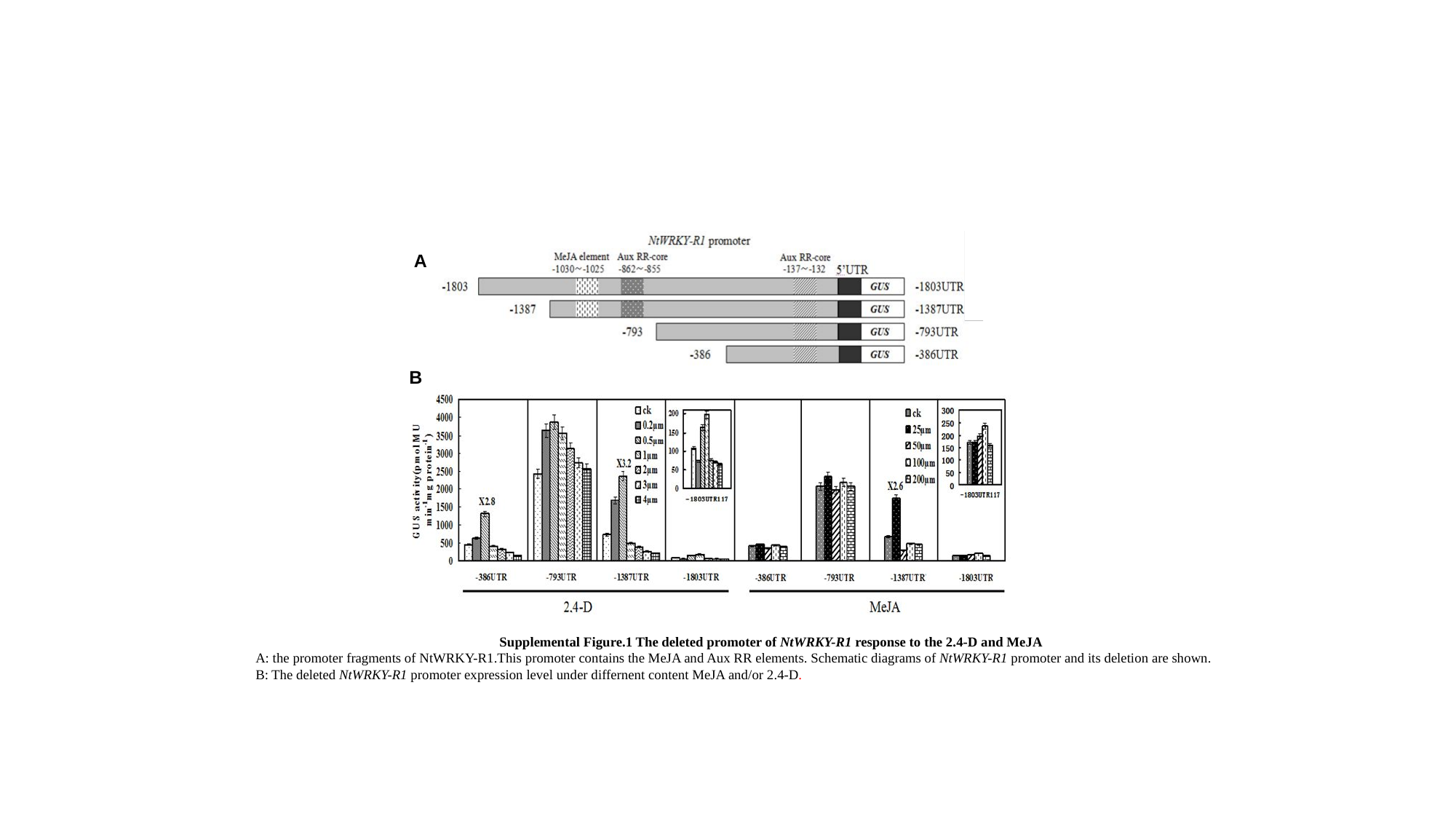

A
B
Supplemental Figure.1 The deleted promoter of NtWRKY-R1 response to the 2.4-D and MeJA
A: the promoter fragments of NtWRKY-R1.This promoter contains the MeJA and Aux RR elements. Schematic diagrams of NtWRKY-R1 promoter and its deletion are shown.
B: The deleted NtWRKY-R1 promoter expression level under differnent content MeJA and/or 2.4-D.
